# Supplementary material for: Identity‐By‐Descent Mapping Using Multi‐Individual IBD With Genome‐Wide Multiple Testing Adjustment
Source: Genet Epidemiol. 2025 Jul 28;49(6):e70015. doi: 10.1002/gepi.70015 (PMC12327188; doi:10.1002/gepi.70015)

## Supplementary materials

Figure S1. Estimated decay parameter $\hat{\alpha}$ for genome-wide IBD mapping tests under different algorithm parameters for multi-individual IBD detection. The left panel shows results using a haplotype length threshold of 2 cM, while the right panel shows results for a 3 cM threshold. The y-axis represents the estimated $\hat{\alpha}$, plotted against the corresponding trimming thresholds used in each test. Results from tests on the simulated sequence data are shown in black, and results from tests on the simulated SNP array data are shown in red.


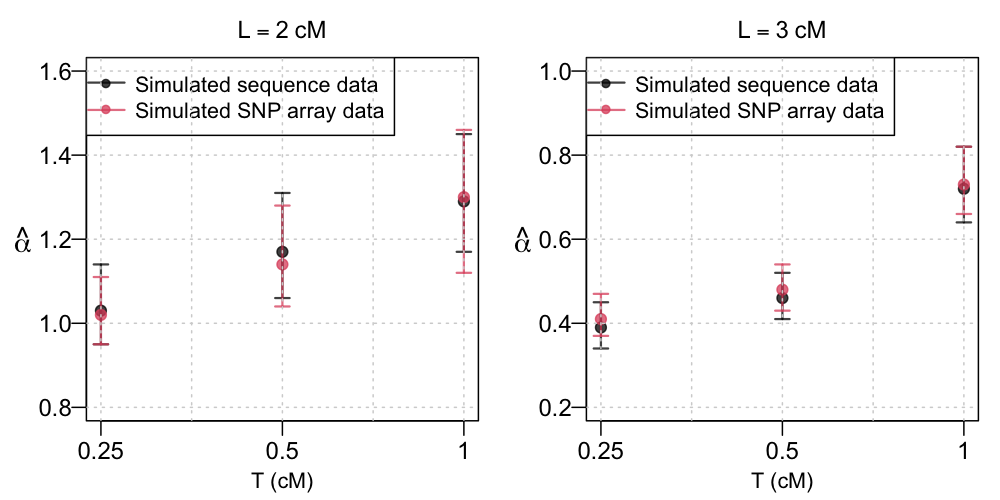


Fig S2. Empirical correlation between test statistics spaced at $d$ cM from genome-wide IBD mapping tests with phenotypes simulated under the null hypothesis on simulated sequence data, using different haplotype length thresholds ($\boldsymbol{L}$) and trimming thresholds ($\boldsymbol{T}$) for multi-individual IBD detection. The 95% confidence interval for the correlation between test statistics spaced at $d$ cM is constructed by taking the 2.5^th^ and 97.5^th^ percentile of the empirical correlation of test statistics spaced at $d$ cM across all bootstrap samples*.* The theoretical correlation is calculated using the corresponding estimated decay parameter $\hat{\alpha}$.


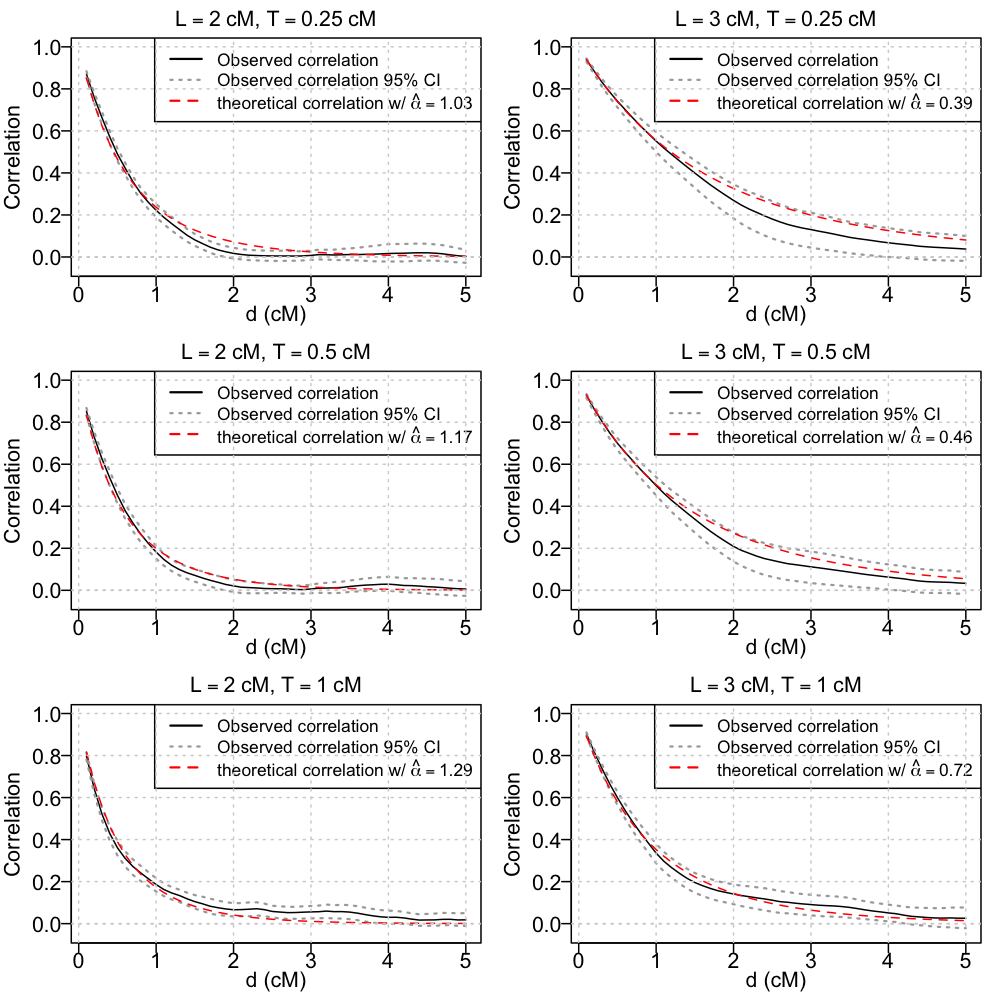


Fig S3. Empirical correlation between test statistics spaced at $d$ cM from genome-wide IBD mapping tests with phenotypes simulated under the null hypothesis on simulated SNP array data, using different haplotype length thresholds ($\boldsymbol{L}$) and trimming thresholds ($\boldsymbol{T}$) for multi-individual IBD detection. The 95% confidence interval for the correlation between test statistics spaced at $d$ cM is constructed by taking the 2.5^th^ and 97.5^th^ percentile of the empirical correlation of test statistics spaced at $d$ cM across all bootstrap samples*.* The theoretical correlation is calculated using the corresponding estimated decay parameter $\hat{\alpha}$.


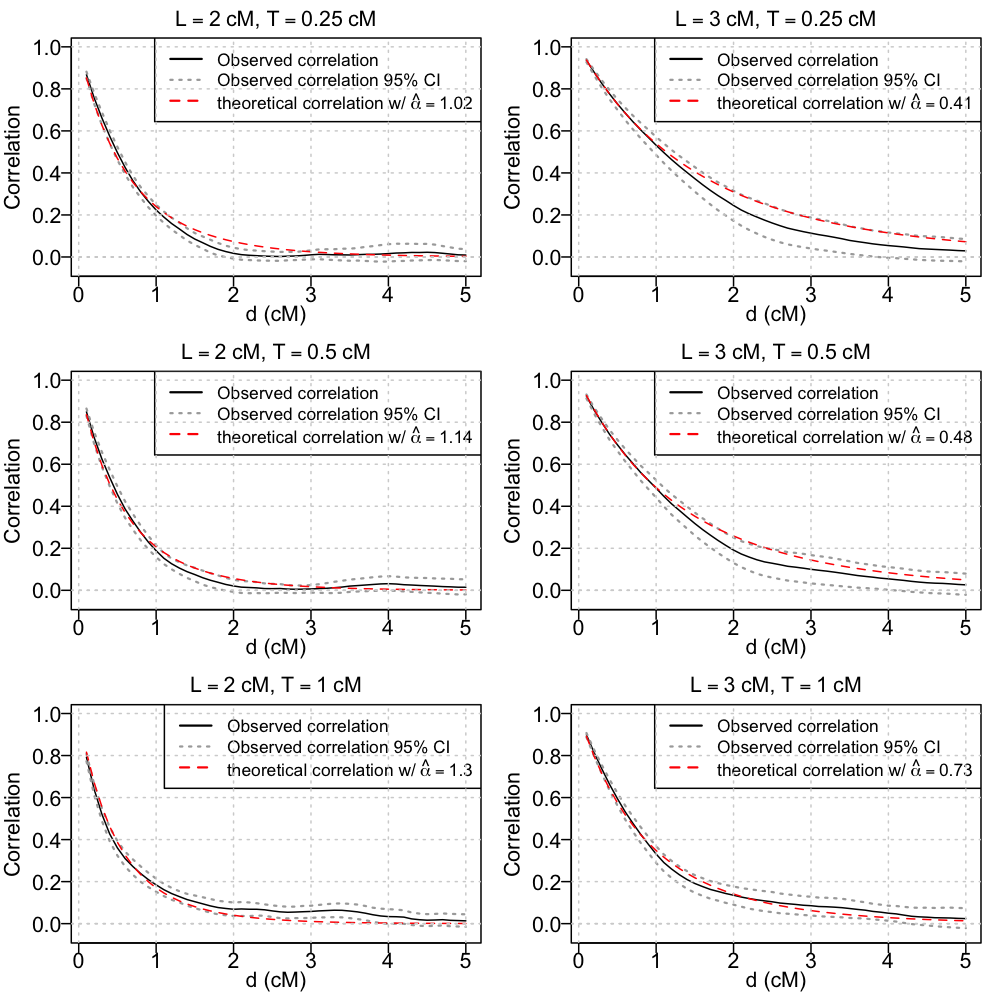


Fig S4. Empirical correlation between test statistics from genome-wide IBD mapping tests with phenotypes simulated under the null hypothesis on the array data of 124,376 White British individuals in the UK Biobank. A haplotype length threshold of 2 cM and a trimming threshold of 0.5 cM were used to detect multi-individual IBD. The empirical correlation and their 95% bootstrap confidence intervals are compared to the theoretical correlation calculated with estimated decay parameter $\hat{\alpha}=1.62$.


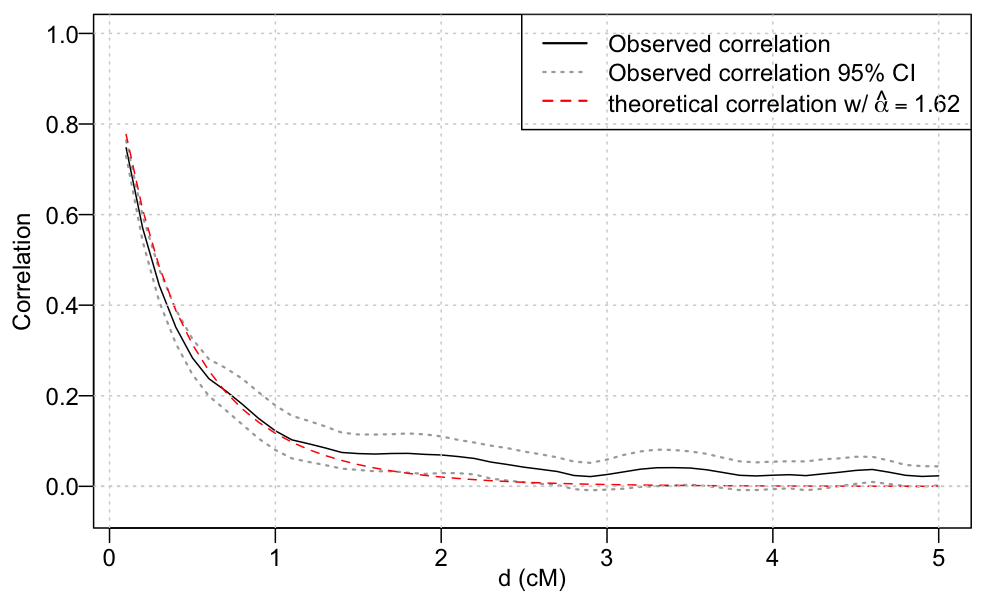


Fig S5. The power of our IBD mapping test for detecting common (>10% MAF), low-frequency (1-10% MAF), rare (0.05-1% MAF), and ultra-rare (<0.05% MAF) causal variants using the Bonferroni correction. On the simulated sequence data, we compared the IBD mapping test to the sequence kernel association test (SKAT). On the simulated SNP array data, we compared the IBD mapping test to the traditional single-variant test used in GWAS. The tick labels on the x-axis indicate the haplotype length threshold ($\boldsymbol{L}$) and the trimming threshold ($\boldsymbol{T}$) used for multi-individual IBD detection in the corresponding IBD mapping test.


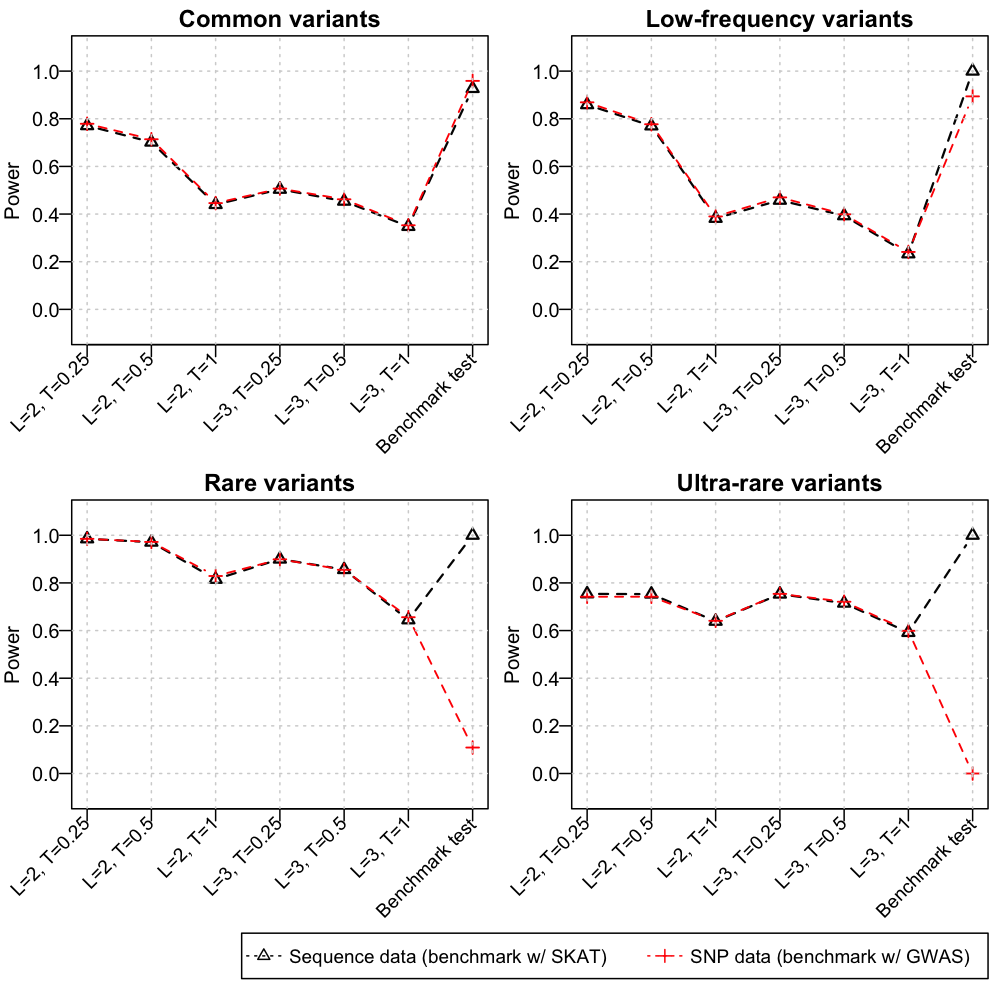


Figure S6. Results of applying the FiMAP test at 1 cM intervals across the genome to systolic blood pressure data from 124k White British individuals in the UK Biobank. Negative log10 transformed p-values are plotted against tested positions on each chromosome along the genome.
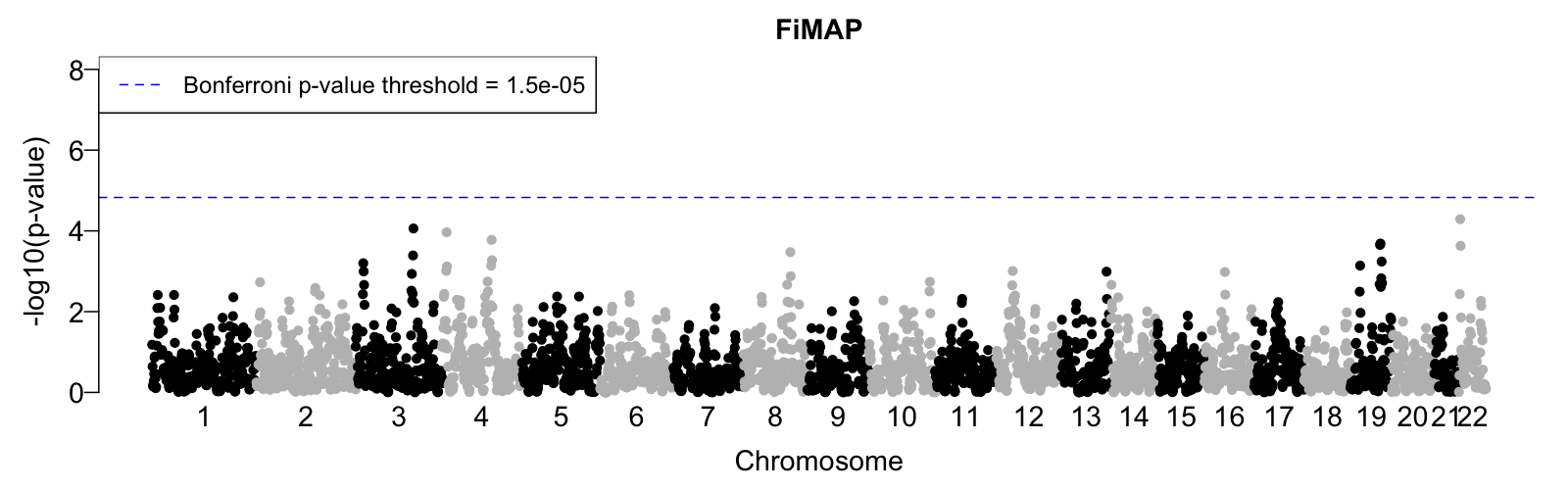

Supplement: Supplementary file 1 — Figure S1: Estimated decay parameter for genome‐wide IBD mapping tests under different algorithm parameters for multi‐individual IBD detection. Figure S2: Empirical correlation between test statistics spaced at cM from genome‐wide IBD mapping tests with phenotypes simulated under the null hypothesis on simulated sequence data, using different haplotype length thresholds ( L ) and trimming thresholds ( T ) for multi‐individual IBD detection. Figure S3: Empirical correlation between test statistics spaced at cM from genome‐wide IBD mapping tests with phenotypes simulated under the null hypothesis on simulated SNP array data, using different haplotype length thresholds ( L ) and trimming thresholds ( T ) for multi‐individual IBD detection. Figure S4: Empirical correlation between test statistics from genome‐wide IBD mapping tests with phenotypes simulated under the null hypothesis on the array data of 124,376 White British individuals in the UK Biobank. Figure S5: The power of our IBD mapping test for detecting common (>10% MAF), low‐frequency (1‐10% MAF), rare (0.05‐1% MAF), and ultra‐rare (<0.05% MAF) causal variants using the Bonferroni correction. Figure S6: Results of applying the FiMAP test at 1 cM intervals across the genome to systolic blood pressure data from 124k White British individuals in the UK Biobank. [file GEPI-49-0-s001.docx]
